# The complete chloroplast genome and phylogenetic analysis of *Christella dentata* (Forssk.) Brownsey & Jermy (Thelypteridaceae)

**DOI:** 10.1080/23802359.2023.2168114

**Published:** 2023-01-25

**Authors:** Guoliang Xu, Changyou Zhang, Shiou Yih Lee, Zhihui Chen, Xiaohui Zeng

**Affiliations:** a Jiangxi Provincial Management Bureau for Jiulian Mountain National Natural Reserve, Longnan, China; b Faculty of Health and Life Sciences, INTI International University, Nilai, Malaysia; c School of Life Science, State Key Laboratory of Biocontrol and Guangdong Provincial Key Laboratory of Plant Resource, Sun Yat-sen University, Guangzhou, China; d Jiangxi Provincial Management Bureau for Jinggang Mountain National Nature Reserve, Ji’an, China

**Keywords:** Chloroplast genome, gene editing, maiden fern, next-generation sequencing, phylogenomics

## Abstract

*Christella dentata* (Forssk.) Brownsey & Jermy (Thelypteridaceae) is endemic to the tropical and subtropical regions of Africa, Asia, and Asia Pacific. In this study, the complete chloroplast genome sequence of *C. dentata* was assembled using next-generation sequencing data. The complete chloroplast genome was 151,662 bp in length and had a typical quadripartite structure, which consisted of a small single-copy region (21,776 bp) and a large single-copy region (82,624 bp) that were separated by a pair of inverted repeats (23,631 bp each). A total of 131 genes were predicted, including 89 protein coding (CDS), 34 tRNA, and eight rRNA genes. The overall GC content of the chloroplast genome was 42.48%. Based on the concatenated shared unique CDS sequence dataset, phylogenetic analysis using both the maximum-likelihood and the Bayesian inference methods revealed that *C. dentata* is placed within Thelypteridaceae and is closely related to *Christella appendiculata*. Such genetic information would be useful for studies on the evolution pattern in ferns. The availability of chloroplast genome sequence for the species also paves the way to resolving the complicated relationship among members of *Christella*.

Thelypteridaceae is widely distributed in the tropical and subtropical regions of the world, with a few species found in temperate regions, especially in Asia (Fawcett and Smith 2021). It is considered as one of the largest families of ferns that are morphologically and ecologically highly diverse, consisting of more than 20 genera and more than 1000 species (Fawcett and Smith 2021). *Christella* H.Lév. (1915) is currently recognized as a distinct genus in Thelypteridaceae, which can be distinguished from other sister genera by its unique characteristic of having thick, elongate, blunt unicellular hairs on the stalks of the sporangia (PPG I 2016). *Christella dentata* (Forssk.) Brownsey & Jermy (1973), also known as the soft/maiden fern, is characterized by its soft and hairy fronds with oblong secondary pinnae and its short-creeping rhizomes, which are unique features useful for species identification (Figure 1; de Lange 2022). Based on literature, the species was first named *Polypodium dentatum* Forssk. (1775) before it was described using other names in later assessments, such as *Christella dentata*, *Cyclosorus dentatus* (Forssk.) Ching (1938), and *Thelypteris dentata* (Forssk.) E.P.St.John (1936) (Brownsey and Perrie 2016). To date, a total of 66 synonyms have been recorded for this fern (POWO 2022), and the name *Christella dentata* is currently accepted by most taxonomist based on the morphological and molecular evidence thus far (Fawcett et al. 2021). The fern is endemic to the tropical and subtropical regions of Africa, Asia, and Asia Pacific, but has also been reported to be an invasive species in many countries (Rebbas et al. 2019). In some regions, the fern is regarded as a food plant and raw material for traditional medicine. Leaf extracts from *C. dentata* have been demonstrated to show pharmacological functions, including antibacterial, antifungal, antihyperglycemic, and antinociceptive properties. It is traditionally used to treat sickness such as diabetes, pain, gout, and rheumatism (Tanzin et al. 2013; Manhas et al. 2018).

**Figure 1.**
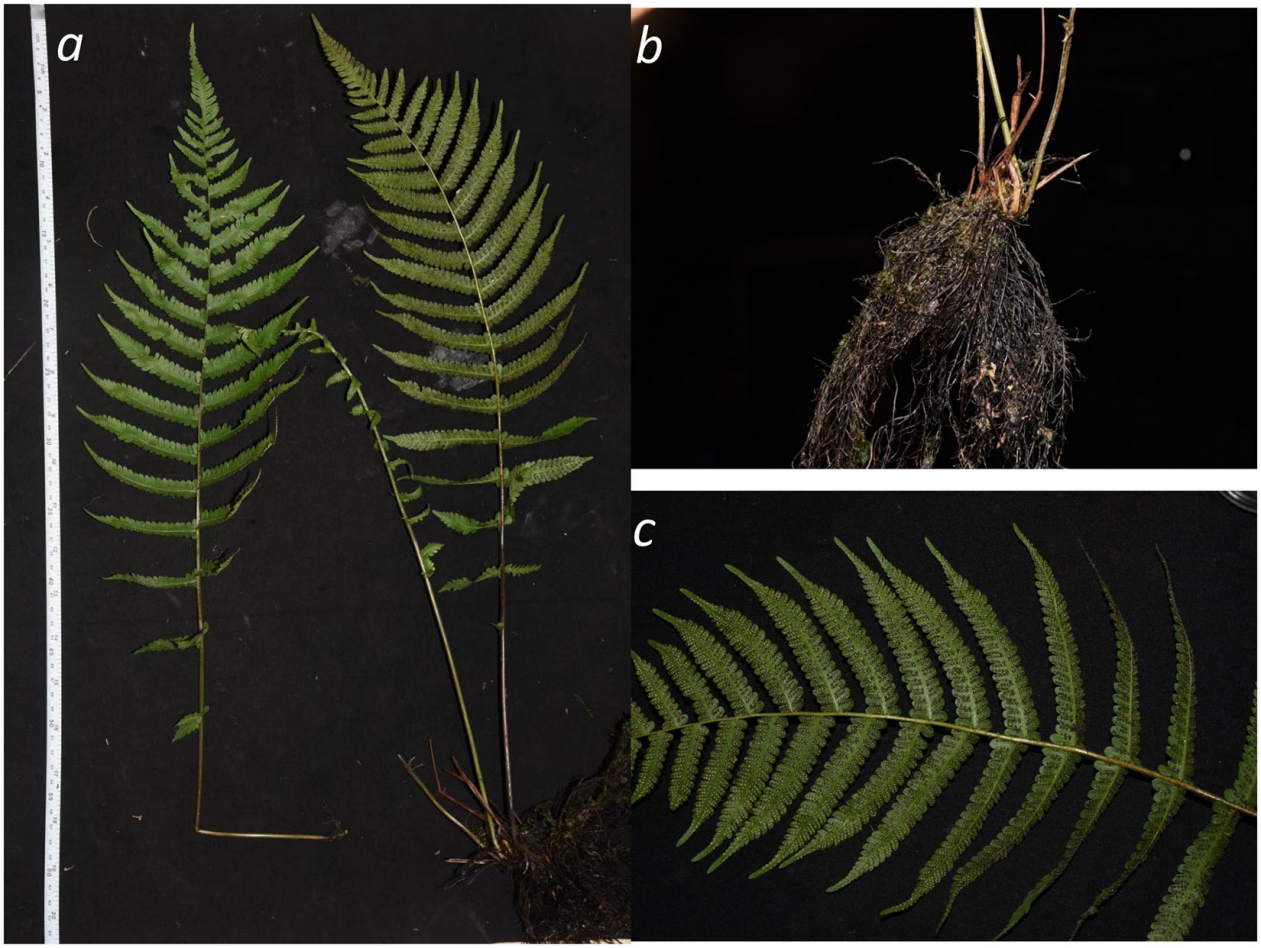
*Christella dentata* growing in its natural habitat. (a) The whole plant; (b) rhizome; (c) leaf. The photo was taken and redacted by SYL and XZ at Mount Jiulian in Longnan County, Jiangxi Province (24°0′37.8″N, 114°0′34.2″E).

Recent studies on the molecular phylogenetic relationship of *C. dentata* used concatenated chloroplast DNA sequences of *rps*4-*trn*S and *trn*L-*trn*F (Almeida et al. 2016), as well as a subset of 407 single-copy nuclear gene loci (Fawcett et al. 2021). With 32 and 14 *Christella* taxa in the systematic analysis alongside with other members of Thelypteridaceae, phylogenetic relationship within *Christella* was resolved using the nuclear DNA dataset, but the molecular placement of *C. dentata* was ambiguous using the chloroplast DNA data. It is believed that chloroplast phylogenomics has the potential to resolve relationships among eupolypod II ferns (Wei et al. 2017) and reveal their evolutionary patterns (Wolf et al. 2011). As an understudied plant that holds great ecological and medicinal value, in this study, the complete chloroplast genome of *C. dentata* was sequenced, which would provide useful information to reveal the genetic identity and molecular position of this fern species among its relatives.

Fresh leaves were collected from an individual of *C. dentata* found on Mount Jiulian in Longnan County, Jiangxi Province (24°0′37.8″N, 114°0′34.2″E). A voucher specimen of the sample has been deposited in the Biological Herbarium of Jiangxi Provincial Management Bureau for Jiulian Mountain National Natural Reserve (contact person: Guoliang Xu; e-mail: 29589268@qq.com) under the collection number JXJLS0001234. Total genomic DNA was extracted from fresh leaves using a modified cetyltrimethyl ammonium bromide (CTAB) method (Doyle and Doyle 1987) and later quantified using a Qubit™ 4 Fluorometer (Fisher Scientific, Waltham, MA). Then, a 350-bp library was constructed and sequenced (paired end, 150 bp) on an Illumina Novaseq platform (Illumina, San Diego, CA). Approximately, 7 GB raw data were obtained and used as input into the NOVOPlasty 4.2 (Dierckxsens et al. 2017) pipeline for plastome assembly. The *rbc*L gene sequence of *C. dentata* (GenBank accession number: MT974507) was set as the seed sequence. Genes were annotated using both CpGAVAS (Liu et al. 2012) and GeSeq v2.03 (Tillich et al. 2017), simultaneously. The annotated chloroplast genome was manually verified and corrected by comparing to the published chloroplast genome of *Cyclosorus interruptus* (GenBank accession number: MN599066; Ramekar, Choi, Cheong, et al. 2020). The chloroplast genome and the genes that were difficult to be annotated were visualized using CPGView (https://www.1kmpgcn/cpgview/). The chloroplast genome sequence was deposited in the NCBI GenBank database under the accession number OM001014.

The minimum and average coverage of the assembled chloroplast genome were 105× and 417×, respectively (Supplementary Figure 1). The complete chloroplast genome of *C. dentata* was 151,662 bp and consisted of a typical quadripartite structure, which is similar to other seed plants (Jansen and Ruhlman 2012). The chloroplast genome contained two 23,631-bp inverted repeat regions that are separated by an 82,624-bp large single-copy region and a 21,776-bp small single-copy region (Figure 2). A total of 131 genes were predicted, including 89 protein coding (CDS), 34 tRNA, and eight rRNA genes. Among them, 16 genes contain two exons, while four genes contain three exons (Supplementary Figure 2), while the gene structure of the trans-splicing gene, *rps*12 was identified (Supplementary Figure 3). Events of gene editing were identified in 41 CDS genes, which is a common phenomenon in ferns (Wolf et al. 2011). The overall GC content was 42.48%.

**Figure 2.**
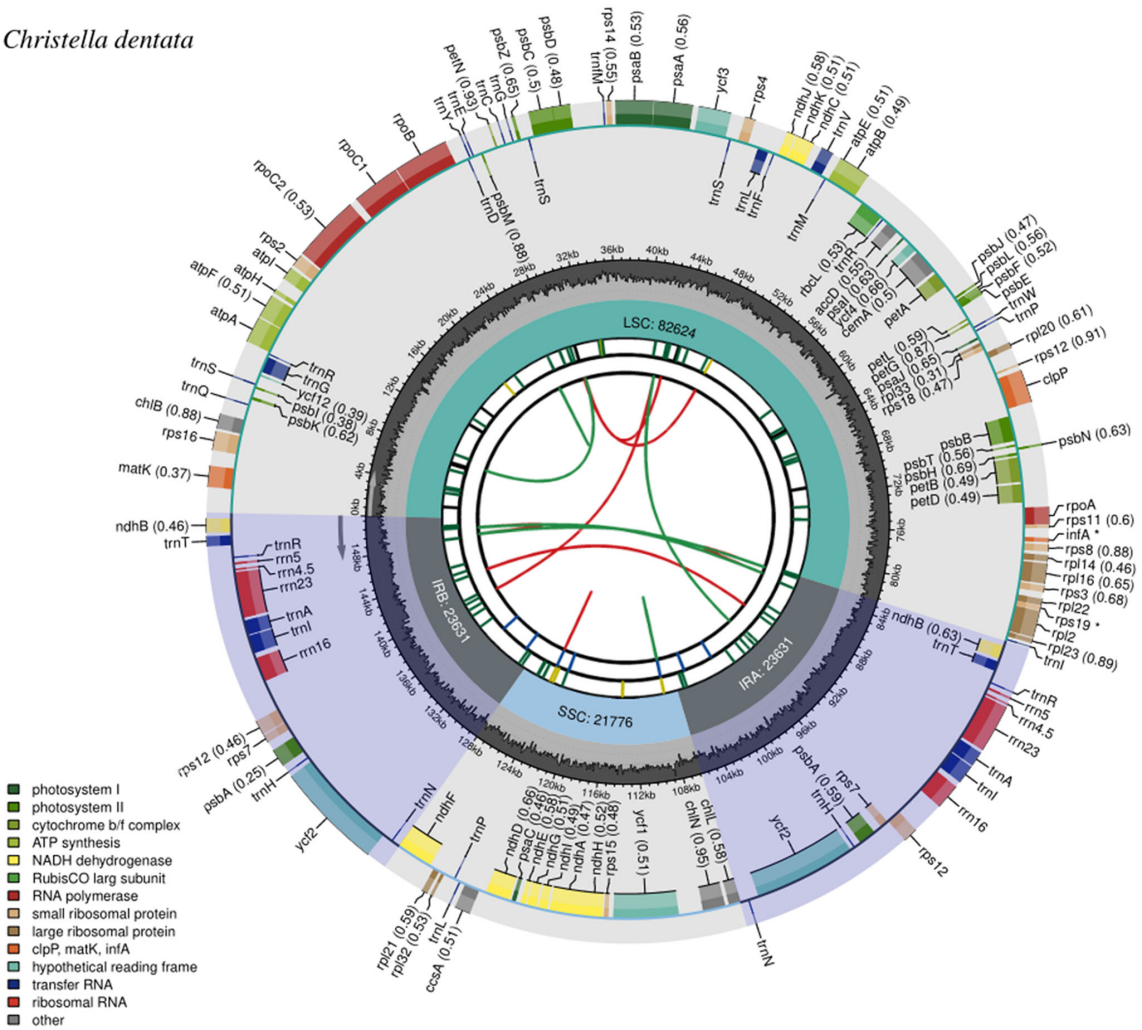
Chloroplast genome map of *Christella dentata.* From the center outward, the first track shows the dispersed repeats, in which the Forward (D) and Palindromic (P) repeats are connected with red and green arcs. The second track shows the long tandem repeats as short blue bars. The third track shows the short tandem repeats or microsatellite sequences as short bars with different colors that correspond to their repeat unit size: Black: complex repeat; green: repeat unit size = 1; yellow: repeat unit size = 2; purple: repeat unit size = 3; blue: repeat unit size = 4; orange: repeat unit size = 5; red: repeat unit size = 6. The small single-copy, inverted repeat, and large single-copy regions are shown on the fourth track. The GC content along the genome is plotted on the fifth track. The genes are shown on the sixth track, while the optional codon usage bias is displayed in the parenthesis after the gene name. Genes are color-coded by their functional classification (bottom left corner), while the transcription directions for the inner and outer genes are clockwise and anticlockwise, respectively.

**Figure 3.**
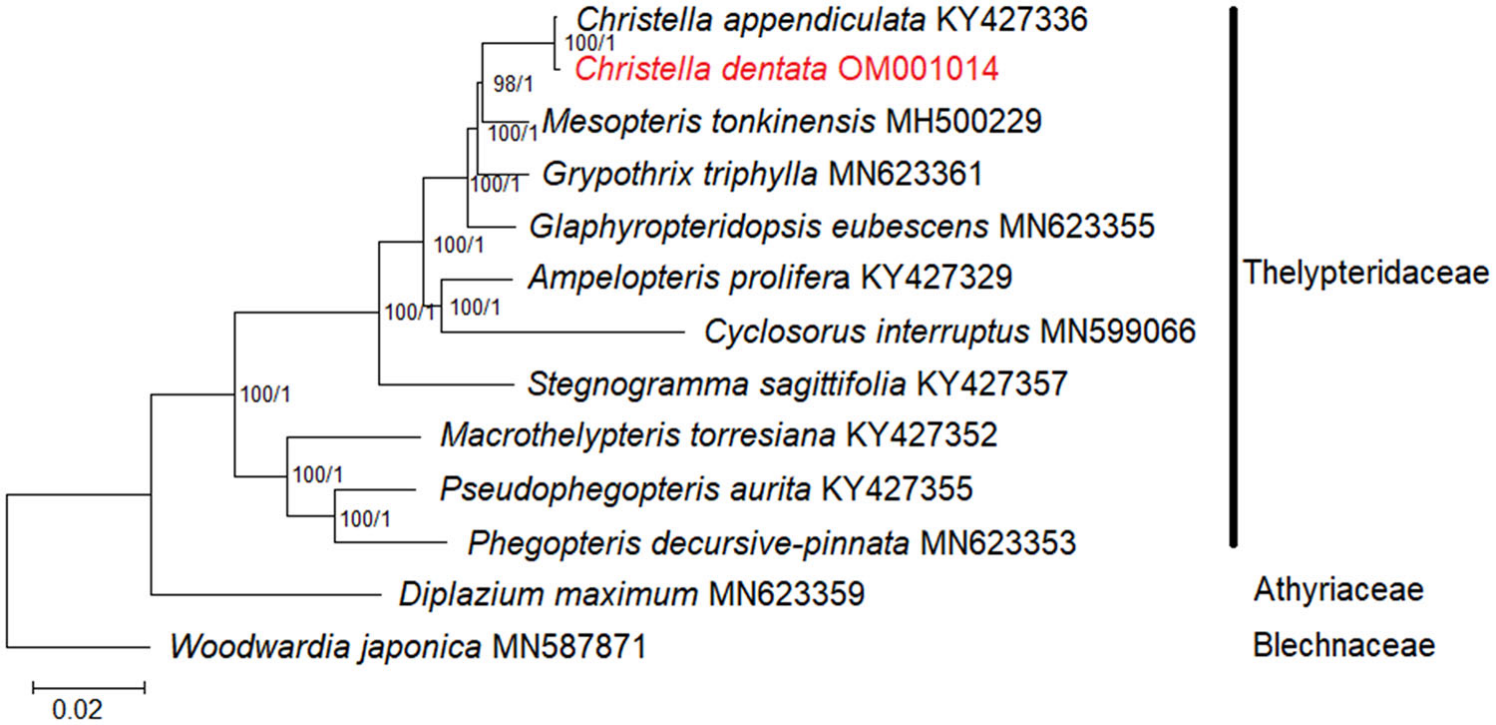
Phylogenetic analysis based on concatenated 82 shared unique CDS in the plastid genome of 13 species of Thelypteridaceae. *Diplazium maximum* (Athyriaceae; GenBank accession number: MN623359) and *Woodwardia japonica* (Blechnaceae; GenBank accession number: MN587871) are included as outgroups (Wei et al. 2017; Liu et al. 2020; Ramekar, Choi, Kwak, et al. 2020). Values of bootstrap support and posterior probability for each branch nodes are as indicated.

In order to reveal the phylogenetic position of *C. dentata*, the complete chloroplast genome sequences of 11 fern species under Thelypteridaceae were selected for phylogenetic analysis (Wei et al. 2017; Liu et al. 2020; Ramekar, Choi, Kwak, et al. 2020). Prior to phylogenetic tree reconstruction, 82 shared unique CDS sequences of each species were extracted, aligned, and concatenated using BEDTools (Quinlan 2014). Phylogenetic analysis was carried out using the maximum-likelihood (ML) and the Bayesian inference (BI) methods using RAxML v8.2.12 (Stamatakis 2014) and MrBayes v3.2 (Ronquist et al. 2012), respectively. For ML analysis, a general-time reversible (GTR) with gamma distribution (+G) (=GTR + G) nucleotide substitution model coupled with 1000 bootstrap replicates was selected; while for BI analysis, a mixed substitution type and a 4 × 4 (4 by 4) nucleotide substitution model were selected, and the Markov chain Monte Carlo was conducted for 2,000,000 generations, with readings sampled every 100 cycles. Two closely related species, *Diplazium maximum* (Athyriaceae; GenBank accession number: MN623359; Liu et al. 2020) and *Woodwardia japonica* (Blechnaceae; GenBank accession number: MN587871; Ramekar, Choi, Kwak, et al. 2020) were included as outgroups. As both the ML and BI tree displayed similar topologies, only the ML tree is shown (Figure 3). Based on current circumscription, the phylogenetic tree is well-resolved; *C. dentata* is closely related to *Christella appendiculata* under strong branch support values (bootstrap support ≥75%, posterior probability ≥0.90). Similar to the finding based on the plastid dataset by Almeida et al. (2016), *C. dentata* is placed under the christelloid clade that contains genera *Glaphyropteridopsis* and *Mesopteris*. The chloroplast genome of ferns is important information to fern evolution and phylogeny as they would undergo high levels of gene editing, as demonstrated in *C. dentata*, a trait that is rare for seed plants (Wolf et al. 2011). Despite the complicated taxonomic status of *Christella*, phylogenetic inference based on the complete chloroplast genome sequence could offer more resolution compared to short plastid sequences, aiding future taxonomic revisions of *Christella* at the species level.

## Supplementary Material

Supplemental Material

Supplemental Material

Supplemental Material

## Data Availability

The plastid genome sequence data in this study are openly available in GenBank of NCBI at https://www.ncbi.nlm.nih.gov/ with the accession number OM001014. Raw sequencing reads used here have been deposited in the SRA database of NCBI under accession number SRR17418362. The associated ‘BioProject’ and ‘Bio-Sample’ numbers are PRJNA793617 and SAMN24566055, respectively.
