# Supplementary figures and images for: The complete chloroplast genome and phylogenetic analysis of Christella dentata (Forssk.) Brownsey & Jermy (Thelypteridaceae)
Source: Mitochondrial DNA B Resour. 2023 Jan 25;8(1):181–5. doi: 10.1080/23802359.2023.2168114 (PMC9879191; doi:10.1080/23802359.2023.2168114)

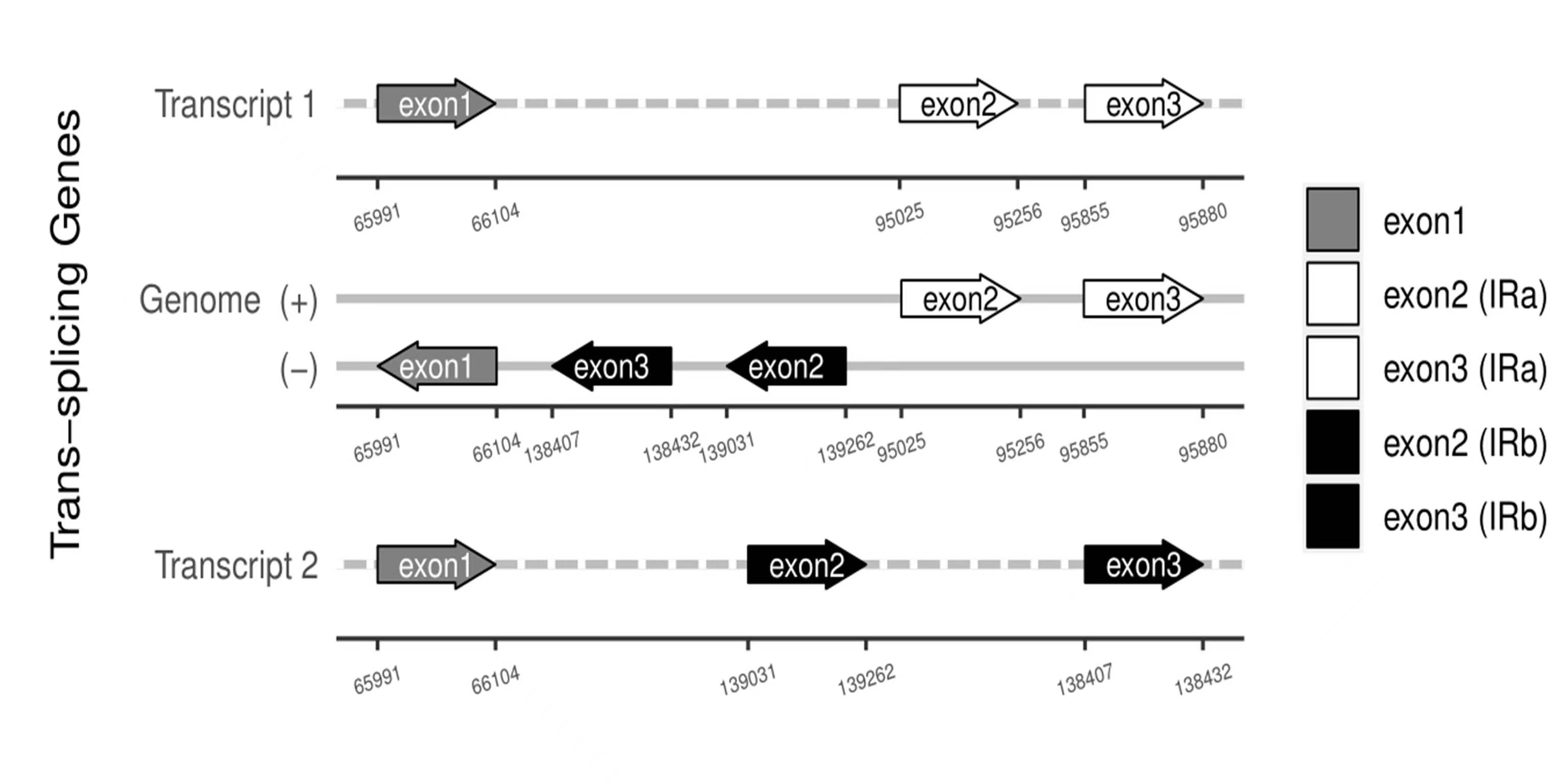

Supplement: Supplemental Material [file TMDN_A_2168114_SM4145.jpg]

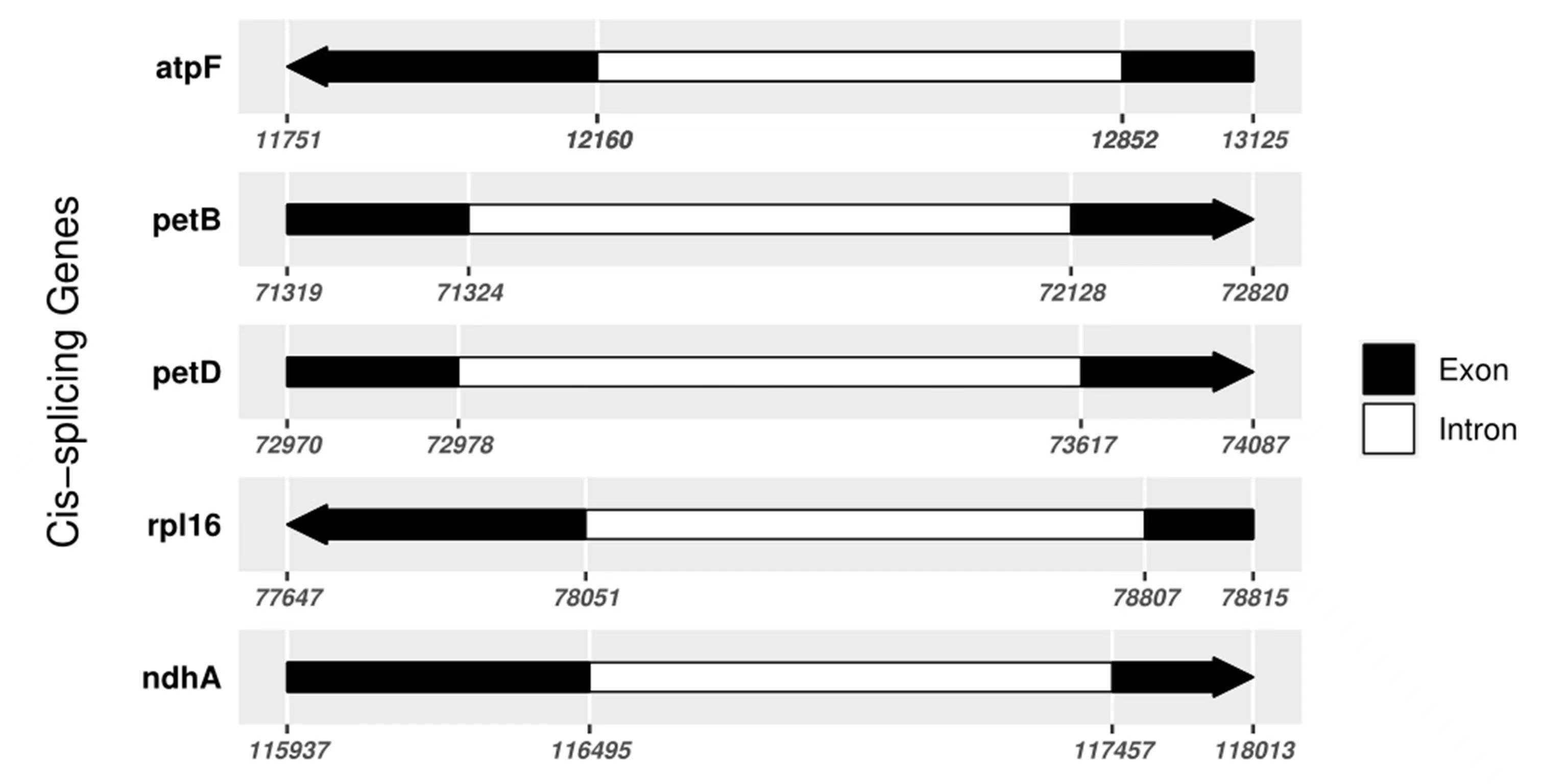

Supplement: Supplemental Material [file TMDN_A_2168114_SM4144.jpg]

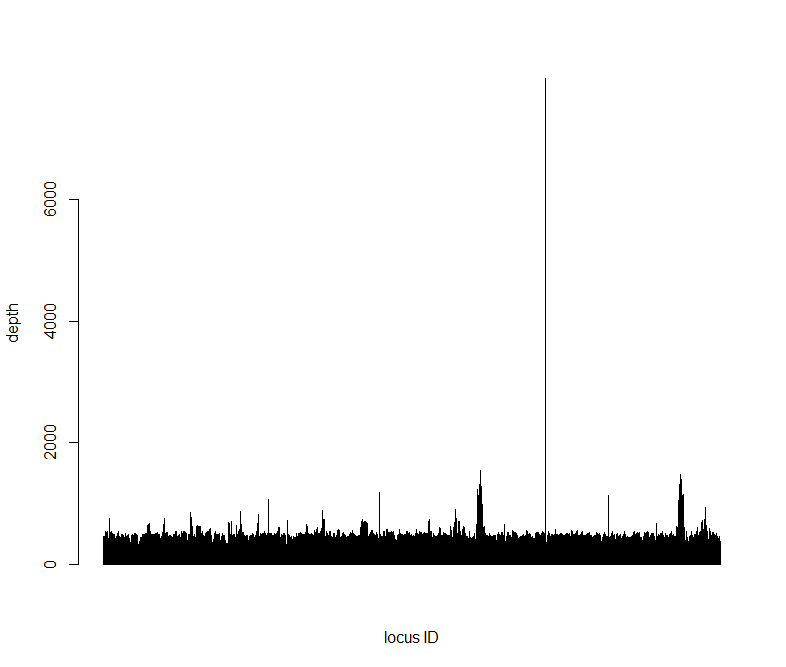

Supplement: Supplemental Material [file TMDN_A_2168114_SM4143.tiff]
